# Brassinosteroid Coordinates with ROS, Auxin and Gibberellin to Promote Mesocotyl Elongation and Deep-Sowing Tolerance in Maize

**DOI:** 10.3390/cimb47080668

**Published:** 2025-08-18

**Authors:** Yahui Wang, Ying Li, Yuze Ma, Xiaolin Wu, Wei Wang, Hui Liu, Xiaoming Li

**Affiliations:** 1 State Key Laboratory of High-Efficiency Production of Wheat-Maize Double Cropping, College of Life Sciences, Henan Agricultural University, Zhengzhou 450046, China; 2 International Education College, Henan Agricultural University, Zhengzhou 450046, China; 3 College of Agronomy, Shandong Agricultural University, Tai’an 271018, China

**Keywords:** brassinosteroid, mesocotyl elongation, reactive oxygen species, auxin, gibberellin, maize

## Abstract

Mesocotyl elongation is the key determinant of deep-sowing tolerance in maize. Sowing at an appropriate depth allows the seedling to exploit water and nutrients stored in deeper soil layers, thereby enhancing its ability to withstand drought and other abiotic stresses. Mesocotyl elongation is regulated by the phytohormones brassinosteroid (BR), auxin (IAA), gibberellin (GA), and reactive oxygen species (ROS). However, whether and how BR coordinates IAA, GA, and ROS to control mesocotyl elongation in maize remains unclear. Here, we demonstrated that BRs orchestrate ROS, IAA, and GA signaling to remodel cell-wall metabolism in mesocotyl cells, promote cell elongation, and, consequently, strengthen deep-sowing tolerance. BR promoted mesocotyl elongation through multiple routes: (1) decreasing the contents of cell-wall components (hemicellulose, cellulose, and pectin); (2) activating cell-wall-loosening enzymes (cellulase, pectinase, and acidic xylanase); and (3) disturbing ROS homeostasis by elevating superoxide dismutase (SOD) activity. Combined treatments of BR with either IAA or GA further enhanced mesocotyl elongation in a concentration-dependent manner. In deep-sowing trials (15 cm), application of BR alone or in combination with IAA or GA markedly increased mesocotyl length and emergence rate, thereby improving deep-sowing tolerance. Our work indicated that BR integrated ROS, IAA, and GA signals to restructure the cell wall and derived mesocotyl cell elongation, providing both theoretical insights and practical strategies for breeding maize varieties with enhanced deep-sowing tolerance.

## 1. Introduction

Maize (*Zea mays* L.) is a globally vital crop, valued for its broad environmental adaptability and high yield potential. In many high-altitude, arid, and semi-arid regions, deep sowing is a widely adopted cultivation strategy [1]. The length of the mesocotyl is a critical factor determining whether deeply sown maize can emerge successfully. Sowing at appropriate depths enables seeds to access moisture and nutrients in deeper soil, as well as more stable temperatures, thereby helping seedlings penetrate the soil surface, improving emergence rates, and enhancing tolerance to drought and other adverse conditions [2]. The mesocotyl, as an essential structure in grass seedlings, links the coleoptilar node to the basal part of the seedling. Horizontally, the mesocotyl is composed of the vascular cylinder, cortex, and epidermis; vertically, it consists of the meristematic, elongating, and mature zones from top to bottom [3]. The mesocotyl’s elongation propels the coleoptile upward, enabling seedlings to break through the soil surface. The length of the mesocotyl directly influences seedling emergence capacity. Under deep-sowing conditions, a longer mesocotyl enhances upward growth of the coleoptile, facilitating soil penetration and improving emergence rates. Studies have shown that maize with longer mesocotyls exhibits greater adaptability to deeper sowing depths, utilizes deeper soil moisture, and displays enhanced emergence rate [4,5]. Understanding maize mesocotyl elongation is key to breeding deep-sowing, high-yield varieties under stress.

Mesocotyl elongation is regulated by multiple plant hormones. Studies have shown that brassinosteroid (BR), Auxin (IAA), and gibberellin (GA) are all involved in controlling mesocotyl elongation in maize [2,6,7]. BR is a master regulator of virtually every facet of plant life, orchestrating cell elongation and division [8]. Mutants deficient in BR exhibit dwarfism and shortened primary roots, whereas exogenous application of 24-epibrassinolide (EBR), a bioactive BR, alleviates these symptoms, confirming BR’s essential function in plant development and stress response [9,10,11]. EBR significantly increased the mesocotyl length of maize under a 20 cm deep-sowing stress [12]. Using RNA-seq analysis, the study indicated that EBR promoted maize mesocotyl elongation by modulating a set of genes involved in cell-wall components, regulating enzymes, and hormone signaling, such as IAA and GA [12]. IAA is one of the most important phytohormones in plants, with extremely diverse functions [13,14]. It activates the proton pump (H^+^ -ATPase), loosens the cell wall, and allows water to enter, thereby promoting cell elongation [15,16]. The study showed that application of IAA promotes mesocotyl elongation in maize [17]. Recent work indicated that the transcription factor ZmNAC17 contributed to controlling mesocotyl elongation in maize. The *zmnac17* mutant displayed a markedly shorter mesocotyl than the wild type, a phenotype that correlated with a significant reduction in endogenous IAA content [18]. GA plays crucial roles in promoting cell elongation, breaking seed dormancy, and stimulating fruit development [19]. In maize, exogenous application of gibberellin significantly promotes mesocotyl elongation and enhances seedling emergence [4,20]. These results underscore the pivotal roles of BR, IAA, and GA in governing mesocotyl growth in maize.

Reactive oxygen species (ROS) are byproducts of normal aerobic metabolic processes in plants [21]. ROS can also be generated in other cellular compartments, including peroxisomes and microbodies. NADPH oxidase (RBOH proteins) is a plasma-membrane enzyme that catalyzes the formation of apoplastic superoxide (O_2_^−^), thereby generating ROS signals essential for processes such as cell elongation and stress responses [22,23]. Superoxide radicals (O_2_^−^), precursors to various ROS, can spontaneously or enzymatically convert into H_2_O_2_ via superoxide dismutase (SOD), oxalate oxidase, or polyamine oxidase. ROS can be generated both intracellularly and extracellularly, though their lifespans are extremely short [24]. Among ROS, H_2_O_2_ is the most stable (half-life exceeding 1 millisecond) and is recognized as a critical redox signaling molecule. It can be spontaneously metabolized into H_2_O and O_2_ by class III peroxidases [24]. Despite their cytotoxic potential, previous studies demonstrate that plants utilize ROS as signaling molecules to regulate gene expression during meristem maintenance, organ development, and stress responses [25,26,27], particularly in promoting polysaccharide metabolism [28,29]. Hydroxyl radicals (OH) are hypothesized to participate in polysaccharide cleavage, increasing cell-wall extensibility and enabling cell growth [30,31]. The synergistic regulation of organ development by ROS and hormonal signals has emerged as a new frontier in plant biology. However, the mechanisms by which ROS regulate cell elongation remain poorly understood, and in maize, there have been no reports on ROS-mediated control of mesocotyl elongation. A recent study showed that mutation of *ZmNAC17* results in shorter mesocotyls, possibly associated with elevated ROS levels [18].

Mesocotyl elongation is jointly controlled by the three major hormones BR, IAA, and GA, together with ROS, yet how these signals integrate to drive rapid mesocotyl growth under deep-sowing conditions remains largely unexplored. Using maize as the model, this study elucidated the molecular mechanism by which BR orchestrated ROS and the classical hormonal pathways to promote mesocotyl elongation, thereby improving emergence rate and drought tolerance under deep sowing, providing both theoretical insights and technical support for cultivating maize under drought conditions.

## 2. Materials and Methods

### 2.1. Materials and Growth Conditions

The hybrid maize variety Zhengdan 958 was used as the experimental material. The maize hybrid Zhengdan 958 was developed by Henan Academy of Agricultural Sciences. It was produced by crossing the inbred line Zheng 58 as the female parent with Chang 72 as the male parent. The Zhengdan 958 seeds used in this study were obtained from Henan Yunqiu Seed Company Limited, China. The surface of maize seeds was disinfected with 75% ethanol for 5 min and then rinsed thoroughly with clean water. Then, the seeds were soaked in distilled water for 12 h. After soaking, the seeds were placed embryo-up on a germination tray lined with gauze. The distilled water was added to the germination tray, and plastic wrap was placed on top. The tray was then placed in a 28 °C incubator for 2 days until germination. After germination, the plastic wrap was removed, and the treatment solution was sprayed at an interval of 24 h for a total of 3 times. Then, the seeds were cultured in the dark at 28 °C in the incubator until the 7th day. The mesocotyl length of the etiolated maize seedlings was measured, and phenotypes were photographed.

### 2.2. Treatment Assays

This study systematically evaluated the effects of BR, H_2_O_2_, diphenyleneiodonium (DPI), IAA, and GA_3_ applied alone or in combination with 1 μM BR on the mesocotyl elongation of etiolated maize seedlings. All solutions were prepared with distilled water, and the following protocol was used for every treatment: after water-cultured seeds germinated, the treatment solution was sprayed once every 24 h for a total of three applications; the seeds were then incubated in darkness at 28 °C for 7 d, after which mesocotyl length was measured and phenotypes were photographed. Data were obtained from at least three independent replicates and were presented as mean ± standard deviation (SD). The specific concentrations of each reagent used in this study are as follows: BR concentrations: 0, 0.01, 0.1, 1, and 10 μM (0 μM served as the control); 1 μM BR was selected for subsequent co-treatments. H_2_O_2_ concentrations: 0, 0.25, 0.5, 0.75, 1, and 2 mM (0 mM served as the control); DPI concentrations: 0, 0.4, 1, and 2 μM (0 μM served as the control); IAA concentrations: 0, 1, 10, and 20 μM (0 μM served as the control); GA_3_ concentrations: 0, 0.01, 0.1, 1, and 2 mM (0 mM served as the control). In this study, we used mock treatments as a control for non-specific effects of spraying. For BR, IAA, and GA, which were dissolved in ethanol to prepare stock solutions, we used ethanol-diluted distilled water as the mock treatment. For DPI, which was dissolved in DMSO to prepare the stock solution, we used DMSO-diluted distilled water as the mock treatment. These mock treatments were applied at the same volume and frequency as the experimental treatments to ensure that any observed effects were specifically due to the chemicals of interest rather than the physical act of spraying or the solvent itself.

### 2.3. Paraffin Sections of Mesocotyl

Samples of the mesocotyl from the control group and the treatment group were cut into small segments of 2–3 mm in length and fixed in FAA fixative at 4 °C for 24 h to ensure the integrity of cell morphology. Then, the samples were placed in a dehydration box and dehydrated. After that, they were embedded in paraffin, sectioned, and stained. The prepared sections were observed under a microscope and photographed. Twenty mesocotyl cells were randomly selected from each group of sections, and their lengths were measured using Image J software (v.1.54f). The mean ± SD values were calculated from at least three independent experiments. All images were acquired on an upright fluorescence microscope (Ni-U, Nikon, Tokyo, Japan) at 200× magnification with the following acquisition settings: exposure ≈ 40%, saturation 1.2, gamma 1.0. Sections were stained with 1% toluidine blue. For quantification, images were imported into ImageJ (v.1.54f) and converted to 8-bit grayscale. A scale of 200 µm was calibrated from the embedded scale bar. Cell length was measured manually with the segmented line tool.

### 2.4. Measurement of Cellulose, Hemicellulose, and Total Pectin Contents

Samples of the mesocotyl from the control group, the 1 μM BR group, the 0.75 mM H_2_O_2_ group, the 1 μM BR + 0.75 mM H_2_O_2_ group, the 0.4 μM DPI group, and the 1 μM BR + 0.4 μM DPI group were used to determine the contents of pectin, cellulose, and hemicellulose in the mesocotyl using the pectin, cellulose, and hemicellulose content detection kits from Solarbio Company, Beijing, China. Each group of samples was biologically replicated three times. The experimental operations and the calculation of the contents of each component were carried out according to the instructions of the kits. The product number of the pectin content detection kit is BC1405. The product number of the cellulose content detection kit is BC4285. The product number of the hemicellulose content detection kit is BC4445.

### 2.5. Measurement of Pectinase, Cellulase, and Acidic Xylanase Activities

Samples of the mesocotyl from the control group, the 1 μM BR group, the 0.75 mM H_2_O_2_ group, the 1 μM BR + 0.75 mM H_2_O_2_ group, the 0.4 μM DPI group, and the 1 μM BR + 0.4 μM DPI group were used to determine the activities of pectinase, cellulase, and xylanase in the mesocotyl using the pectinase, cellulase, and xylanase activity detection kits from Solarbio Company, Beijing, China. Each group of samples was biologically replicated three times. The experimental operations and the calculation of enzyme activities were carried out according to the instructions of the kits. The product number of the pectinase activity detection kit is BC2630. The product number of the cellulase activity detection kit is BC2540. The product number of the xylanase activity detection kit is BC2600.

### 2.6. NBT and DAB Staining Assays

DAB Staining Solution Formula: 50 mg of DAB-4HCl was added to 45 mL of distilled water, 25 μL of Tween 20 was added, and then 2.5 mL of 200 mM Na_2_HPO_4_ was added. The mesocotyl was placed in the DAB staining solution and shaken in the dark at 80 rpm for 24 h. Each group of samples was biologically replicated three times. After staining, the mesocotyl was photographed, and the gray value was measured using ImageJ software (v. 1.54f).

NBT Staining Solution Formula: 50 mg of NBT was fully dissolved in 100 mL of Tris buffer (pH 7.4). The mesocotyl was placed in the NBT staining solution and shaken in the dark at 80 rpm for 48 h. Each group of samples was biologically replicated three times. After staining, the mesocotyl was photographed, and the gray value was measured using ImageJ software (v.1.54f).

### 2.7. Measurement of POD and SOD Activities

Samples of the mesocotyl from the control group, the 1 μM BR group, the 0.75 mM H_2_O_2_ group, the 1 μM BR + 0.75 mM H_2_O_2_ group, the 0.4 μM DPI group, and the 1 μM BR + 0.4 μM DPI group were used for DAB staining and NBT staining. The above mesocotyl samples were used to measure the activities of SOD and Peroxidase (POD) using the SOD and POD kits from Solarbio Company, Beijing, China. Each group of samples was biologically replicated three times. The experimental operations and the calculation of enzyme activities were carried out according to the instructions of the kits. The product number of the POD activity detection kit is BC0095. The product number of the SOD activity detection kit is BC0175.

### 2.8. Measurement of Mesocotyl After Deep Sowing of Maize

The hybrid maize variety Zhengdan 958 was used as the experimental material. The surface of maize seeds was disinfected with 75% ethanol for 5 min and then rinsed thoroughly with distilled water. Subsequently, the seeds were soaked in distilled water as the control group, and soaked in 1 μM BR solution as the treatment group for 12 h, respectively. A flowerpot with a depth of 20 cm was used, and the flowerpot was divided into two halves in the middle. One half was used as the CK control group, and the other half was used as the BR treatment group. The soaked seeds were placed at depths of 5 cm, 10 cm, and 15 cm from the soil surface, respectively, and the soil was covered to be level with the edge of the flowerpot. An equal amount of water was poured into each flowerpot, and the flowerpots were placed in a 28 °C greenhouse (28 °C, 60 % relative humidity, and 16,800 lux illumination with a 16 h light/8 h dark photoperiod) for 8 days. The emergence rate, mesocotyl length, etc., were measured, and phenotypic pictures were taken. The deep sowing methods of the combined treatments of 1 μM BR and 10 μM IAA and 1 μM BR and 1 mM GA_3_ were the same as above.

### 2.9. Statistical Analysis

Outliers were identified by box-plot (1.5 × IQR) and confirmed by Z-score (|Z| > 3). The extreme values were winsorized to the 5th/95th percentile. The final n for each group is reported in the figure legends. The Shapiro–Wilk test showed that the data were normally distributed (*p* > 0.05). Homogeneity of variances was assessed using Levene’s test prior to ANOVA or *t*-tests, and the assumption was met (*p* > 0.05). Comparisons between two groups were evaluated using an unpaired two-tailed Student’s *t*-test; significance was indicated as * *p* < 0.05, ** *p* < 0.01, and *** *p* < 0.001. When more than two groups were compared, one-way analysis of variance (ANOVA) followed by Tukey’s honestly significant difference (HSD) test was applied, with statistical significance set at * *p* < 0.05. All statistical analyses were performed in R (version 4.5.1).

To minimize observer bias, all samples used for mesocotyl-length measurements were assigned random numeric codes. Sowing and measurement were performed by different personnel, and the measurers remained unaware of the treatment groups throughout the process, thereby establishing observer blinding.

### 2.10. Principal Component Analysis

Prior to Principal Component Analysis (PCA), data suitability was evaluated. The Kaiser–Meyer–Olkin (KMO) measure was 0.740 (>0.60), and Bartlett’s test of sphericity was significant (*p* < 0.001), indicating adequate correlations among variables for PCA. PCA was performed with R (version 4.5.1). The extraction method was set to Principal Components, using the correlation matrix as the analysis basis. Components were retained according to eigenvalues ≥ 1 (Kaiser criterion). Component interpretation was based on the unrotated component matrix. Loadings with absolute values ≥ 0.50 were considered significant.

### 2.11. Correlation Analysis

Correlation analysis was performed among the differential maize mesocotyl elongation values (treatment minus control) across all treatments. A Pearson correlation heat-map was constructed using the OmicShare online platform (www.omicshare.com/tools; accessed on 10 August 2025). Pairwise Pearson correlation coefficients (r) are displayed in the upper triangle of the matrix. Each coefficient was evaluated for significance with a two-tailed Student’s *t*-test (* *p* < 0.05; ** *p* < 0.01; *** *p* < 0.001; non-significant values are omitted). The color gradient visualizes the strength and direction of each relationship (red; positive; blue; negative).

## 3. Results

### 3.1. BR Regulates Cell-Wall Metabolism to Promote Cell Elongation in Maize Mesocotyls

**Figure 1:**
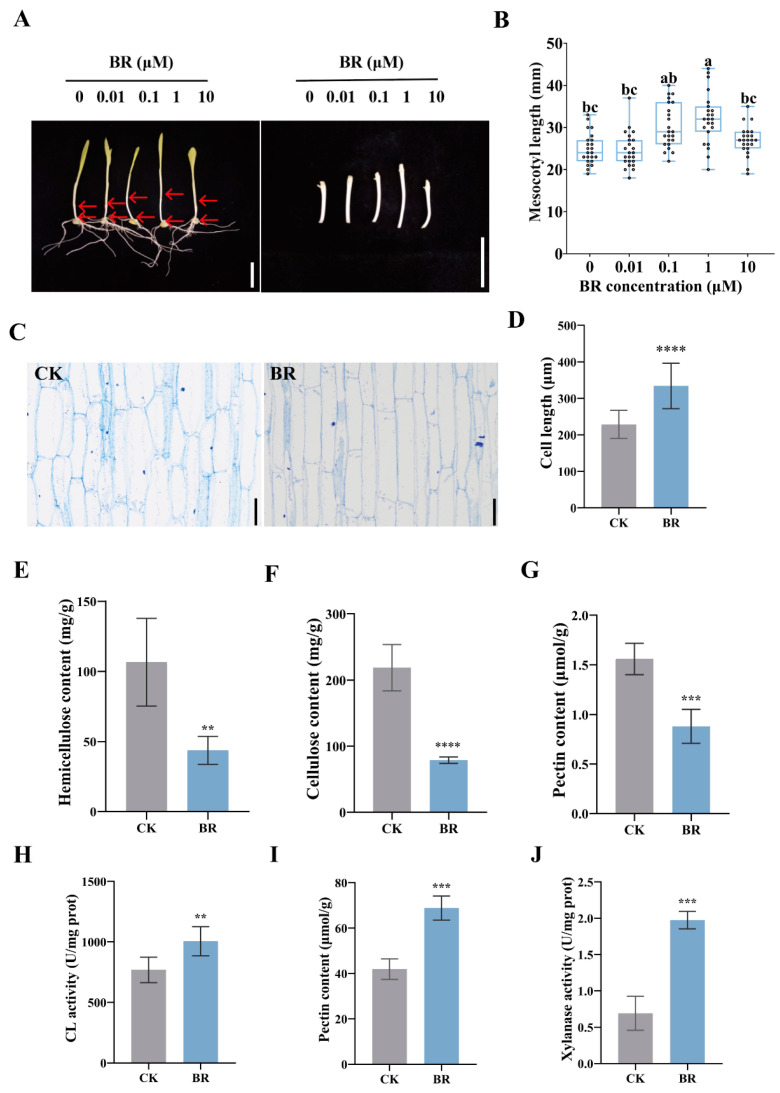
Effects of BR application on the mesocotyl of maize: (**A**) Phenotypes of the whole yellowing seedlings and mesocotyls of Zhengdan 958 maize after spraying BR solutions at concentrations of 0 μM, 0.01 μM, 0.1 μM, 1 μM, and 10 μM following seed germination. In each maize seedling, the mesocotyl is indicated by the upper and lower red arrows. (**B**) Statistical analysis of mesocotyl lengths under different BR treatments (n = 23). Scale bar, 40 mm. Different letters (a, b, c) indicate significant differences among groups after one-way ANOVA followed by Tukey’s HSD test (*p* < 0.05). Any two groups marked with the same letter are not significantly different (*p* ≥ 0.05). (**C**) Longitudinal section diagrams of the fiber structure of the mesocotyls of yellowing maize seedlings in the control group and under 1 μM BR treatment. Scale bar,200 μm. (**D**) Statistical analysis of cell lengths in the mesocotyls of yellowing maize seedlings in the control group and under 1 μM BR treatment. Data are presented as the mean ± SD (n = 20); n represents the number of maize mesocotyl cell. (**E**) Hemicellulose content. (**F**) Cellulose content. (**G**) Total pectin content. (**H**) Cellulase activity. (**I**) Pectinase activity. (**J**) Acidic xylanase activity. CK, the control group. BR, treatment with 1 μM BR. Statistical significance (**D**–**J**) was analyzed using Student’s *t*-test (** *p* < 0.01, *** *p* < 0.001, **** *p* < 0.0001). Data are presented as the mean ± SD (n = 3). n is the biological replicate size; a biological replicate is an independent maize mesocotyl (**B**), or is a single independent experiment (**E**–**J**).

A previous study has reported that BR promotes mesocotyl elongation in maize [12]; however, the effective BR concentration may vary among different maize genotypes. Therefore, for the hybrid Zhengdan 958, we established a BR concentration gradient to identify the most effective dose. The result indicated that exogenous application of BR significantly promoted mesocotyl elongation in maize (Figure 1A,B), and the optimal BR concentration for maximal elongation was 1 μM, achieving a 1.3-fold increase in length compared to the control. These results indicate that exogenous BR treatment effectively enhances mesocotyl elongation, with 1 μM BR selected for subsequent experiments. To elucidate the regulatory mechanism of BR on mesocotyl growth, microscopic analysis of longitudinal sections revealed that BR treatment markedly increased cell elongation (Figure 1C). Cells in the BR-treated group were 1.46-fold longer than those in the control (Figure 1D), suggesting that BR may regulate mesocotyl growth by promoting cell elongation.

Cell-wall components, including pectin, cellulose, and hemicellulose, directly influence cell-wall strength and flexibility [32]. BR-treated mesocotyls exhibited significantly reduced contents of hemicellulose, cellulose, and pectin compared to the control (Figure 1E–G). The hemicellulose content in the BR group was 0.41-fold of the control (Figure 1E), the cellulose was 0.36-fold (Figure 1F), and the total pectin was 0.56-fold (Figure 1G). Pectinases, cellulases, and xylanases degrade cell-wall polysaccharides into monosaccharides or oligosaccharides [33]. Enzyme activity assays showed that BR treatment significantly enhanced cellulase, pectinase, and acidic xylanase activities (Figure 1H–J). The cellulase activity increased 1.31-fold, the pectinase activity 1.59-fold, and the acidic xylanase activity 2.85-fold in the BR-treated group compared to the control. Our results indicate that BR-induced mesocotyl elongation is accompanied by a marked decrease in the abundance of hemicellulose, cellulose, and pectin, coupled with a substantial increase in the activities of their corresponding hydrolytic enzymes. This coordinated weakening of the cell wall likely enhances its extensibility, thereby facilitating rapid cell elongation in the maize mesocotyl.

The above results suggest that exogenous H_2_O_2_ and DPI each promote maize mesocotyl elongation independently, and this effect is further amplified when combined with 1 μM BR. Both treatments act by markedly increasing cell length. These findings demonstrate that mesocotyl elongation is dose-dependently regulated by ROS levels—either a moderate increase (via H_2_O_2_) or a moderate decrease (via DPI) stimulates elongation—and that BR synergistically enhances this response. The results establish the optimal treatment combinations for future studies on the cooperative control of mesocotyl growth by ROS and BR in maize.

### 3.2. BR Synergizes with ROS to Promote Mesocotyl Cell Elongation in Maize

**Figure 2:**
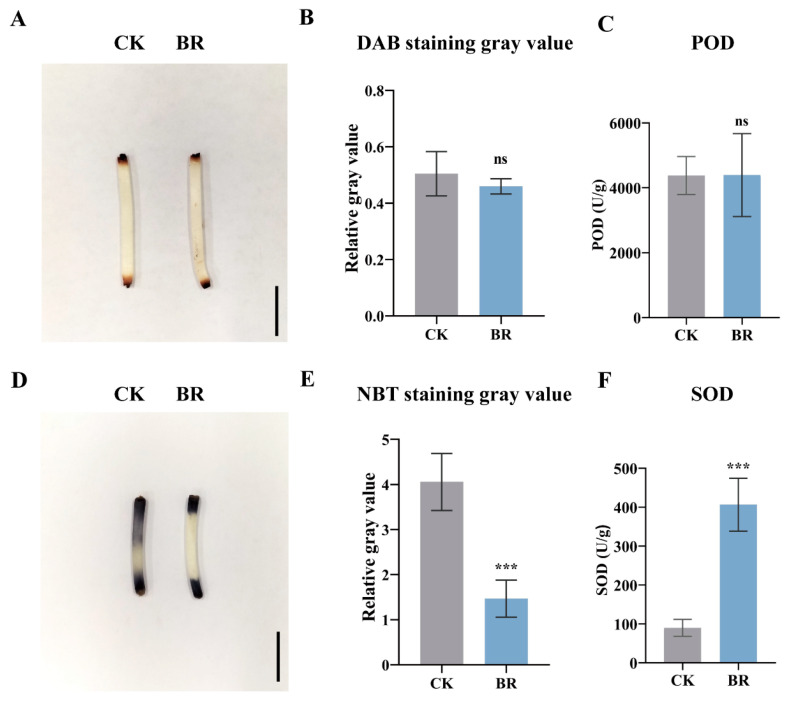
Determination of NBT and DAB staining and POD and SOD activities after BR treatment: (**A**) DAB staining. (**B**) Gray scale analysis of DAB staining. (**C**) POD activity. (**D**) NBT staining. (**E**) Gray scale analysis of NBT staining. (**F**) SOD activity. Scale bar, 1 cm. CK, the control group. BR, treated with 1 μM BR. Statistical significance was analyzed using Student’s *t*-test (*** *p* < 0.001), with ns indicating no significant difference. Data are presented as the mean ± SD (n = 3). n is the biological replicate size; a biological replicate is a single independent experiment (**B**,**C**,**E**,**F**).

Oxidants such as H_2_O_2_ and superoxide anion (O_2_^−^) can depolymerize the cell-wall polysaccharides, weakening wall integrity and altering permeability, which ultimately perturbs normal plant development [34]. Given that BRs are known regulators of cell elongation [12], it remained to be tested whether BR-mediated modulation of ROS levels contributes to this process. DAB staining and NBT staining are effective methods for detecting H_2_O_2_ and O_2_^−^ levels, respectively [35]. Peroxidase (POD) and superoxide dismutase (SOD) are critical enzymes for scavenging ROS. SOD converts O_2_^−^ into H_2_O_2_, while POD further decomposes H_2_O_2_ into H_2_O and hydroxyl radicals (·OH) [24,36]. We focused on the central 1.5 cm region for DAB/NBT staining and grayscale analysis. Results showed no significant difference in DAB staining between the control and BR-treated groups (Figure 2A,B), with no significant difference in POD activity between the control and BR-treated groups (Figure 2C). Whereas NBT staining intensity was notably weaker in the BR-treated group compared to the control (Figure 2D), with significantly lower grayscale values (Figure 2E). Enzyme activity assays revealed that SOD activity in the BR-treated group was significantly higher than that in the control (Figure 2F). BR treatment did not alter H_2_O_2_ accumulation or peroxidase activity but markedly reduced superoxide levels and elevated SOD activity in the mesocotyl. These findings indicate that BR alleviates oxidative stress primarily by enhancing superoxide-scavenging capacity, thus creating a more favorable redox environment for cell elongation.

**Figure 3:**
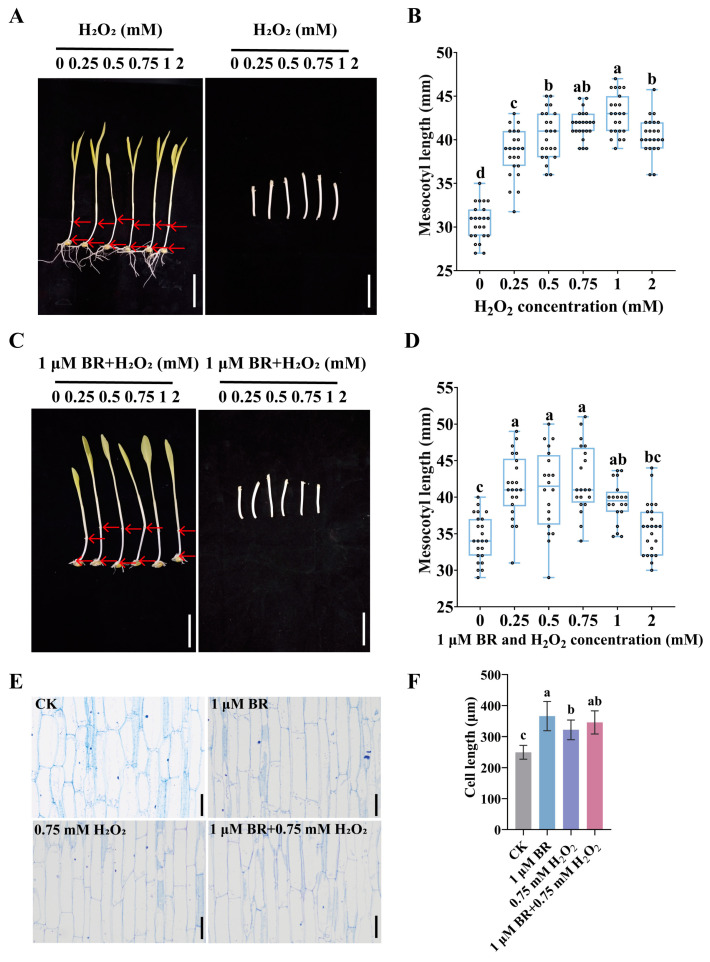
Elongation of the mesocotyl after H_2_O_2_ treatment and combined treatment with BR and H_2_O_2_: (**A**) Phenotypes of the yellowing maize mesocotyls after H_2_O_2_ treatment. Scale bar, 40 mm. In each maize seedling, the mesocotyl is indicated by the upper and lower red arrows. (**B**) Statistical analysis of mesocotyl lengths under different H_2_O_2_ treatments (n = 23). Different letters (a, b, c, d) indicate significant differences among groups after one-way ANOVA followed by Tukey’s HSD test (*p* < 0.05). Any two groups marked with the same letter are not significantly different (*p* ≥ 0.05). (**C**) Phenotypes of maize mesocotyls after combined treatment with 1 μM BR and different concentrations of H_2_O_2_. Scale bar, 40 mm. In each maize seedling, the mesocotyl is indicated by the upper and lower red arrows. (**D**) Statistical analysis of mesocotyl length under the treatment of 1 μM BR and different concentrations of H_2_O_2_ (n = 23). Different letters (a, b, c) indicate significant differences among groups after one-way ANOVA followed by Tukey’s HSD test (*p* < 0.05). Any two groups marked with the same letter are not significantly different (*p* ≥ 0.05). (**E**) Longitudinal section diagrams of the fiber structure of maize mesocotyls in the control group and under different treatments. Scale bar, 200 μm. (**F**) Statistical analysis of cell lengths in the mesocotyls of yellowing maize seedlings in the control group and under different treatments (n = 20); n represents the number of maize mesocotyl cell. Different letters (a, b, c) indicate significant differences among groups after one-way ANOVA followed by Tukey’s HSD test (*p* < 0.05). Data are presented as the mean ± SD (n = 20). n is the biological replicate size; a biological replicate is an independent maize mesocotyl (**B**,**D**).

To determine whether ROS were involved in regulating maize mesocotyl elongation, we used H_2_O_2_, as a stable ROS, for treatment. The results showed that, in the presence of H_2_O_2_, mesocotyl length was significantly greater than in the control group (Figure 3A,B). As H_2_O_2_ concentration changed, mesocotyl length first increased and then decreased. The maximum mesocotyl length was observed at 1 mM H_2_O_2_, which was 1.41 times that of the control group (Figure 3B). These results indicate that exogenous H_2_O_2_ promotes mesocotyl elongation within a certain concentration range. Combined treatment with BR and H_2_O_2_ resulted in significantly longer mesocotyls compared to the control group. Mesocotyl length initially increased with H_2_O_2_ concentration but peaked at 0.75 mM H_2_O_2_ (Figure 3C,D). Based on comprehensive experimental results, subsequent experiments used 1 μM BR and 0.75 mM H_2_O_2_. Longitudinal section observation showed that BR and H_2_O_2_ treatments significantly increased cell length compared to the control (Figure 3E). Cell lengths in the treated groups were 1.47, 1.29, and 1.38 times that of the control (Figure 3F), indicating that exogenous BR and H_2_O_2_ promote mesocotyl elongation.

**Figure 4:**
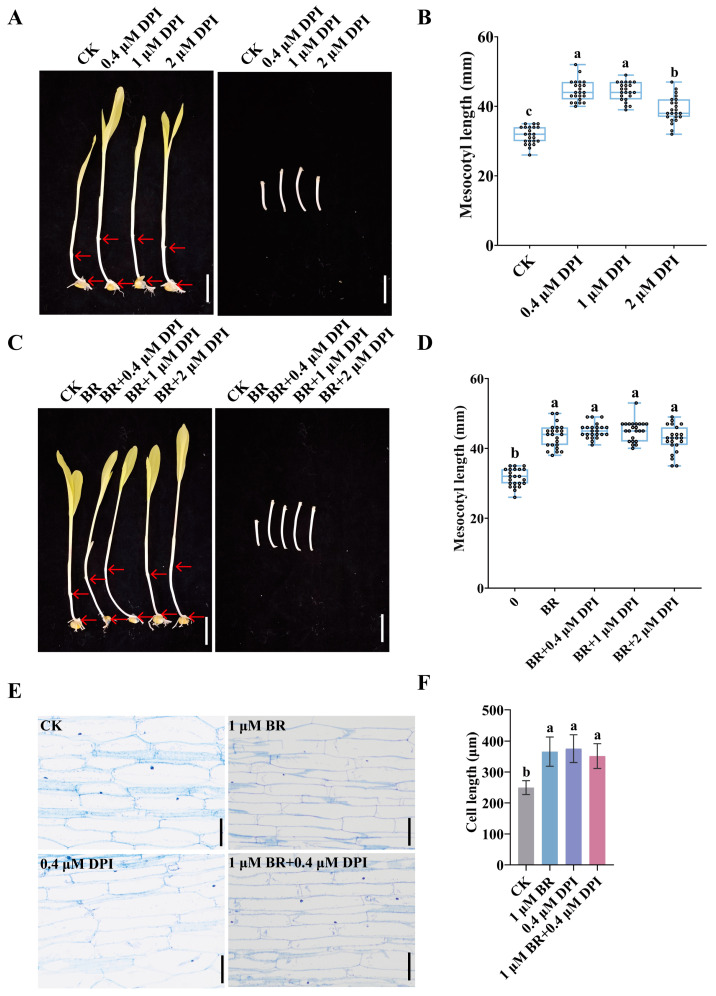
Elongation of mesocotyls after DPI treatment and synergistic treatment with BR and DPI: (**A**) Phenotypes of etiolated maize mesocotyls after DPI treatment. Scale bar, 40 mm. In each maize seedling, the mesocotyl is indicated by the upper and lower red arrows. (**B**) Statistical analysis of mesocotyl lengths under different concentrations of DPI treatment (n = 23). (**C**) Phenotype of mesocotyls in etiolated maize seedlings after synergistic treatment with 1 μM BR and different concentrations of DPI. Scale bar, 40 mm. In each maize seedling, the mesocotyl is indicated by the upper and lower red arrows. (**D**) Statistical analysis of mesocotyl length under the treatment of 1 μM BR and different concentrations of DPI (n = 23). (**E**) Longitudinal section diagrams of fiber structures in mesocotyls of etiolated maize seedlings in the control group and different treatment groups. Scale bar, 200 μm. (**F**) Statistical analysis of cell lengths in mesocotyls of etiolated maize seedlings in the control group and different treatment groups (n = 20); n represents the number of maize mesocotyl cell. Data are presented as the mean ± SD (n = 20). Different letters (a, b, c) in (**B**,**D**,**F**) indicate significant differences among groups after one-way ANOVA followed by Tukey’s HSD test (*p* < 0.05). Any two groups marked with the same letter are not significantly different (*p* ≥ 0.05). n is the biological replicate size; a biological replicate is an independent maize mesocotyl (**B**,**D**).

DPI is a well-known reagent that blocks the activity of the NADPH oxidase, thereby inhibiting ROS production [37]. Next, we employed DPI to assess the impact of reduced ROS levels on maize mesocotyl elongation. DPI treatment promoted mesocotyl elongation compared to the control, with the mesocotyl length first increasing and then decreasing as DPI concentration increased. The maximum mesocotyl length was observed at 0.4 μM DPI (Figure 4A), which was 1.41-fold longer than the control (Figure 4B). When BR and DPI were applied synergistically, the mesocotyl length was significantly longer than that of the control. The trend of first increasing and then decreasing was also observed with increasing DPI concentration, peaking at 0.4 μM DPI (Figure 4C,D). Subsequent experiments selected 1 μM BR and 0.4 μM DPI for further analysis, resulting in a mesocotyl length 1.43-fold longer than the control (Figure 4D). Longitudinal section microscopy revealed that BR and DPI treatment significantly increased cell length compared to the control (Figure 4E). The cell lengths in the treated groups were 1.47-fold, 1.5-fold, and 1.41-fold of the control, respectively (Figure 4F). However, compared with BR treatment alone, the additional application of BR and DPI did not significantly increase mesocotyl length (Figure 4C–F). These findings indicate that exogenous application of BR and DPI promotes mesocotyl elongation. Reducing H_2_O_2_ levels with DPI increases mesocotyl length but fails to enhance BR’s promoting effect on mesocotyl elongation.

### 3.3. BR and ROS Decrease Cell-Wall Components by Enhancing Loosening Enzyme Activity in the Maize Mesocotyl

**Figure 5:**
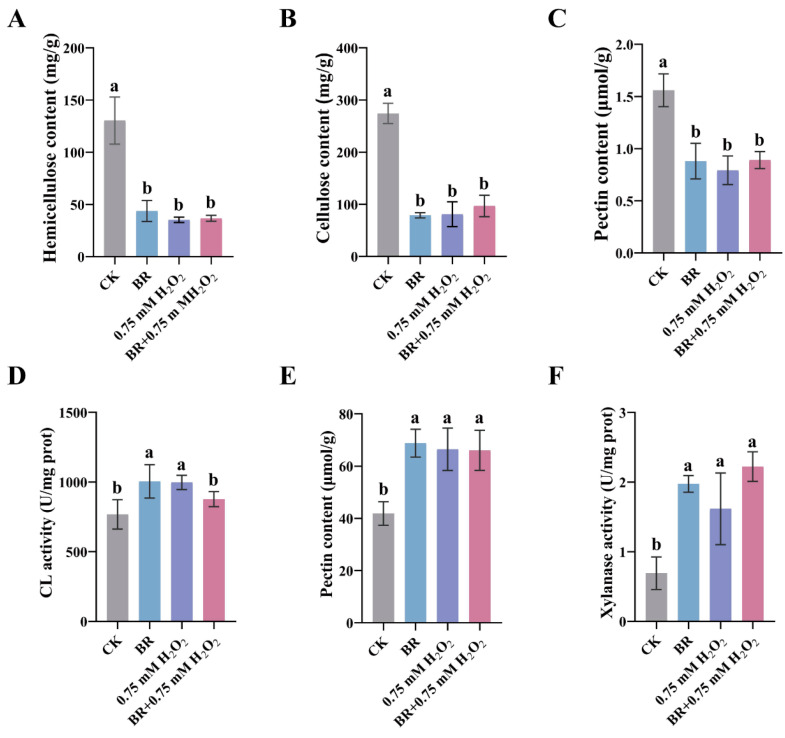
Determination of cell-wall component contents and related enzyme activities in mesocotyl cells after synergistic treatment with BR and H_2_O_2_: (**A**) Hemicellulose content. (**B**) Cellulose content. (**C**) Total pectin content. (**D**) Cellulase activity. (**E**) Pectinase activity. (**F**) Acidic xylanase activity. CK, control group. BR, treatment with 1 μM BR. Different letters (a, b) indicate significant differences among groups after one-way ANOVA followed by Tukey’s HSD test (*p* < 0.05). Any two groups marked with the same letter are not significantly different (*p* ≥ 0.05). Data (**A–F**) are presented as the mean ± SD (n = 3). n is the biological replicate size; a biological replicate is a single independent experiment (**A**–**F**).

To determine whether ROS are involved in the regulation of cell-wall components, we quantified the major polysaccharides after treatment with BR, 0.75 mM H_2_O_2_, or their combination; the contents of hemicellulose, cellulose, and total pectin in the mesocotyl were significantly lower than those in the control group. (Figure 5A–C). These results suggest that exogenous application of BR and H_2_O_2_ may promote mesocotyl elongation by reducing cell-wall strength, facilitating water absorption, and cell turgor-driven expansion. Cellulase, pectinase, and acidic xylanase activities were significantly enhanced in the presence of H_2_O_2_ (Figure 5D–F).

**Figure 6:**
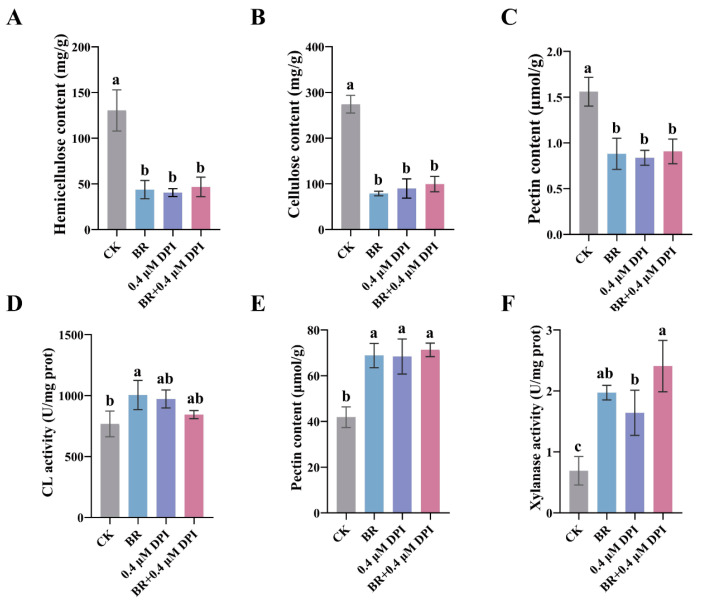
Determination of cell-wall component contents and related enzyme activities in the mesocotyl after synergistic treatment with BR and DPI: (**A**) Hemicellulose content. (**B**) Cellulose content. (**C**) Total pectin content. (**D**) Cellulase activity. (**E**) Pectinase activity. (**F**) Acidic xylanase activity. CK, control group. BR, treated with 1 μM BR. Different letters (a, b, c) indicate significant differences among groups after one-way ANOVA followed by Tukey’s HSD test (*p* < 0.05). Any two groups marked with the same letter are not significantly different (*p* ≥ 0.05). Data are presented as the mean ± SD. Data (**A**–**F**) are presented as the mean ± SD (n = 3). n is the biological replicate size; a biological replicate is a single independent experiment (**A**–**F**).

We examined the cell-wall polysaccharide contents after DPI-mediated ROS suppression; exogenous application of BR, 0.4 μM DPI, or their combination significantly reduced the contents of hemicellulose, cellulose, and total pectin in the mesocotyl compared to the control (Figure 6A–C). However, compared with either treatment alone, the combined BR and DPI application caused no change in the contents of the three substances (Figure 6A–C). This suggests that BR and DPI may promote mesocotyl elongation by reducing cell-wall rigidity, facilitating cell water uptake and expansion. Analysis of cellulase, pectinase, and acidic xylanase activities revealed that, in the presence of DPI, pectinase and acidic xylanase activities were significantly enhanced, while cellulase activity showed no obvious change (Figure 6D–F). Combined treatment with BR and DPI showed no difference in the activities of the three enzymes compared with either treatment alone (Figure 6D–F). These results indicated that lowering H_2_O_2_ levels with DPI did not potentiate BR’s regulation of cell-wall composition or cell-wall-degrading enzyme activities.

Both exogenous H_2_O_2_ and DPI mediated suppression of endogenous ROS function to reduce the contents of hemicellulose, cellulose, and pectin while simultaneously enhancing the activities of cellulase, pectinase, and acidic xylanase, thereby weakening cell-wall rigidity and promoting elongation of the mesocotyl. BR and ROS jointly promote maize mesocotyl elongation by modulating cell-wall-loosening enzyme activities and decreasing cell-wall polysaccharide contents. These findings establish a link between BR and ROS in regulating cell-wall remodeling and provide new experimental evidence for the crosstalk between hormonal and oxidative signaling in controlling plant cell elongation.

### 3.4. BR Coordinates with IAA and GA to Promote Mesocotyl Cell Elongation in Maize

**Figure 7:**
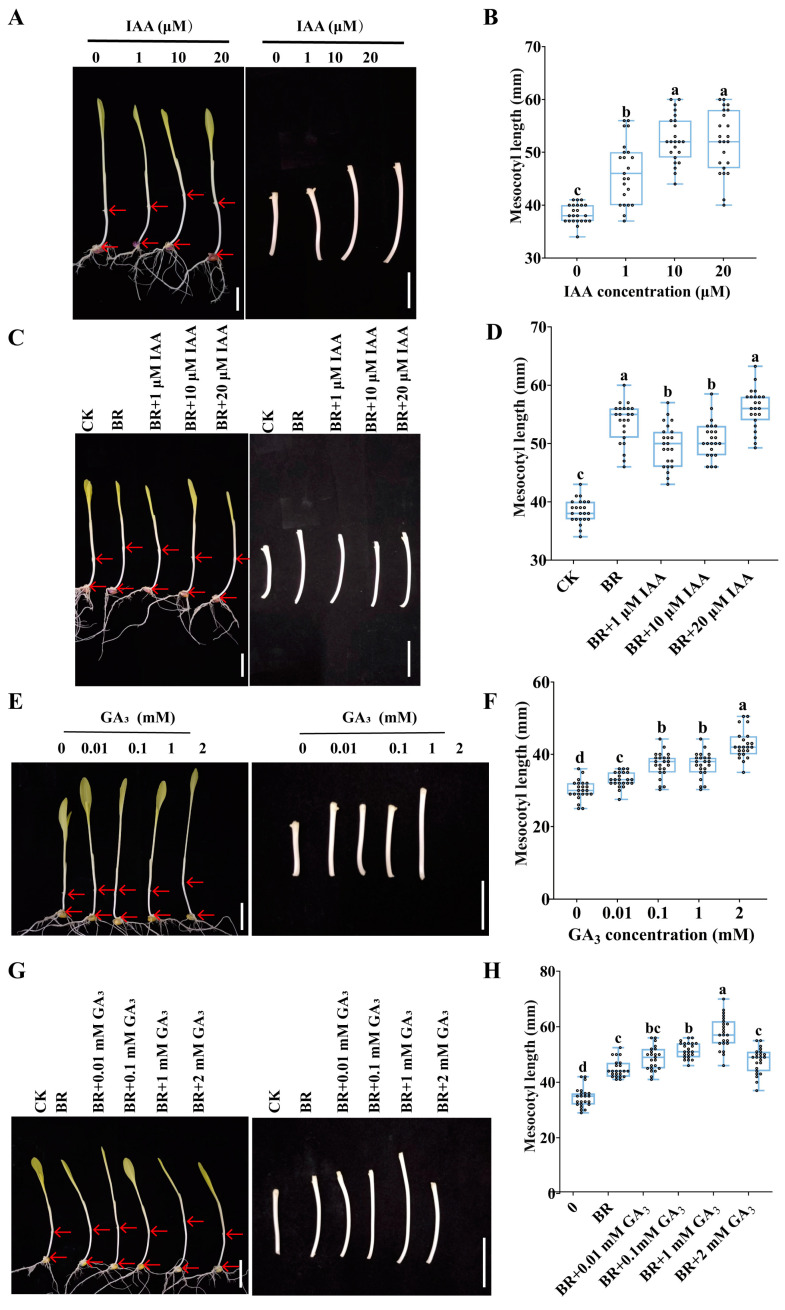
Elongation of mesocotyls after synergistic treatment of BR with IAA or GA_3,_ respectively: (**A**) Phenotype of etiolated maize mesocotyls after IAA treatment. Scale bar, 40 mm. (**B**) Statistical analysis of mesocotyl lengths under different concentrations of IAA treatment (n = 23). (**C**) Phenotype of mesocotyls of etiolated maize seedlings after synergistic treatment with 1 μM BR and different concentrations of IAA. Scale bar, 40 mm. (**D**) Statistical analysis of mesocotyl lengths after treatment with 1 μM BR and different concentrations of IAA (n = 23). (**E**) Phenotype of etiolated maize mesocotyls after GA_3_ treatment. Scale bar, 40 mm. (**F**) Statistical analysis of mesocotyl lengths under different concentrations of GA_3_ treatment (n = 23). (**G**) Phenotype of mesocotyls of etiolated maize seedlings after synergistic treatment with 1 μM BR and different concentrations of GA_3_. Scale bar, 40 mm. (**H**) Statistical analysis of mesocotyl lengths after treatment with 1 μM BR and different concentrations of GA_3_ (n = 23). Different letters (a, b, c, d) in (**B**,**D**,**F**,**H**) indicate significant differences among groups after one-way ANOVA followed by Tukey’s HSD test (*p* < 0.05). Any two groups marked with the same letter are not significantly different (*p* ≥ 0.05). n is the biological replicate size; a biological replicate is a single independent experiment (**B**,**D**,**F**,**H**). In each maize seedling, the mesocotyl is indicated by the upper and lower red arrows (**A**,**C**,**E**,**G**).

For the maize cultivar used in this study, Zhengdan 958, we performed an IAA dose–response experiment to identify the optimal concentration for promoting mesocotyl elongation. The results showed that mesocotyl length increased markedly with rising IAA concentrations, reaching its maximum when the IAA concentration was 10 µM or 20 µM (Figure 7A–B), indicating that exogenous IAA application within a certain concentration range promoted mesocotyl elongation. To determine whether combined treatments of BR with IAA result in even greater enhancement of mesocotyl elongation, we conducted an experiment and found that BR and IAA synergistic treatment resulted in significantly longer mesocotyls than the control, showing a steady upward trend with increasing IAA concentration, whereas combined treatment with BR and IAA had no significant difference compared with the single treatment with BR (Figure 7C–D). Next, we used GA_3_, a highly bioactive form of GA, to examine its regulation of maize mesocotyl elongation. GA_3_ exhibited a concentration-dependent stimulatory effect, with maximal promotion achieved at 2 mM (Figure 7E–F), indicating that exogenous GA application promoted mesocotyl elongation. BR and GA_3_ synergistic treatment led to a first-increase-then-decrease trend in mesocotyl length as GA_3_ concentration increased. When the GA concentration reached 1 mM, the combined GA and BR treatment yielded the most pronounced promotive effect (Figure 7G–H). This suggests that exogenous BR and GA application within a specific concentration range synergistically enhances mesocotyl elongation, with the combined treatment showing a more pronounced promoting effect than BR alone.

Exogenous BR, IAA, and GA each promote maize mesocotyl elongation independently within a certain concentration range. When BR is paired with GA, the stimulatory effect on elongation is markedly amplified; in contrast, co-application with IAA does not confer any additional benefit over BR alone. These findings not only illuminate the dose-dependent synergistic mechanisms between BR and either IAA or GA within the hormonal signaling network but also offer a practical and precise hormone-blend strategy for optimizing maize emergence under deep-sowing conditions.

We performed PCA to reduce dimensionality and visualize the overall relationships among the differential maize mesocotyl elongation values (treatment minus control) across the different treatments. Two components were extracted, jointly accounting for 79.417% of the total variance (Table S1). The unrotated component matrix was used for interpretation; loadings with an absolute value > 0.50 were considered significant (Table S2). PC1 explained 56.893% of the total variance, while PC2 accounted for an additional 22.523% (Table S1). IAA, BR + H_2_O_2_, BR, BR + IAA, BR + GA, GA, and DPI contents were the major contributors to PC1, whereas BR + DPI and DPI contents were mainly associated with PC2 (Table S2). These patterns indicate that BR-regulated antioxidant capacity is the dominant factor underlying the overall physiological variation. The PCA clearly resolved the major patterns underlying the differential mesocotyl elongation responses. PC1 captured the dominant axis of physiological variation, driven by the combined effects of IAA, BR, GA, and H_2_O_2_, and their interactions, while PC2 separated treatments involving BR + DPI and DPI alone, pointing to a distinct role of NADPH-oxidase inhibition. These two components explain nearly 80% of the variance, demonstrating that the key hormonal and oxidative regulators identified here largely dictate the observed differences in maize mesocotyl elongation across treatments. Pearson correlation analysis was applied to quantify and graphically confirm the inter-trait associations. A Pearson correlation heat-map was constructed to visualize the pairwise relationships among the differential maize mesocotyl elongation values (treatment minus control) across all treatments (Figure S1). Strong positive correlations (r > 0.85) were observed between IAA and BR + H_2_O_2_ (r = 0.93), BR and IAA (0.89), and BR and BR + H_2_O_2_ (r = 0.86) (Figure S1). These multivariate analyses provide an integrated view of the relationships among the measured parameters and support the conclusion that BR synergistically orchestrates maize mesocotyl elongation with other phytohormones and reactive oxygen species.

### 3.5. BR Synergizes with IAA and GA to Promote Maize Deep-Sowing Tolerance

**Figure 8:**
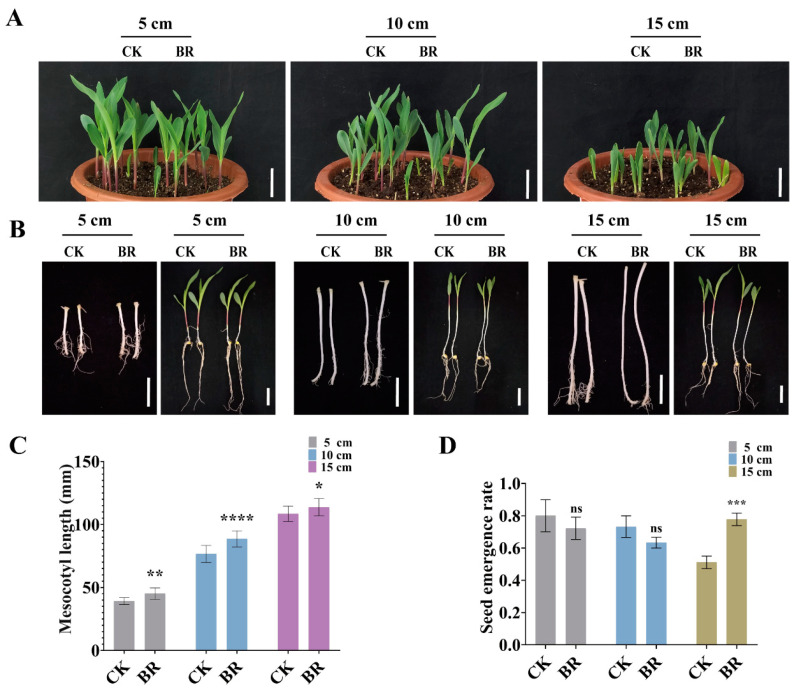
Mesocotyl length and emergence rate treated with BR. (**A**) Ground phenotypes of Zhengdan 958 maize sown at depths of 5 cm, 10 cm, and 15 cm. Scale bar, 5 cm. CK, control group. BR, 1 μM BR treatment. (**B**) Mesocotyl phenotypes of Zhengdan 958 maize sown at depths of 5 cm, 10 cm, and 15 cm. Scale bar, 3 cm. CK, control group. BR, 1 μM BR treatment. (**C**) Statistics of mesocotyl length at sowing depths of 5 cm, 10 cm, and 15 cm. Data are presented as the mean ± SD (n > 14). (**D**) Emergence rate at sowing depths of 5 cm, 10 cm, and 15 cm. Statistical significance (**C** and **D**) was analyzed using Student’s *t*-test (* *p* < 0.05, ** *p* < 0.01, *** *p* < 0.001, **** *p* < 0.0001), with ns indicating no significant difference. Data are presented as the mean ± SD. Data are presented as the mean ± SD (n = 3). n is the biological replicate size; a biological replicate is an independent maize mesocotyl under deep-sowing (**C**), or is a single pot (**D**).

Under deep-sowing conditions, the elongation ability of the mesocotyl is a key factor determining whether seedlings can successfully emerge from the soil. To verify the effectiveness of exogenous application of BR in improving the deep-sowing tolerance of maize, this study set up experiments with different sowing depths (5 cm, 10 cm, and 15 cm), and compared the performance of maize seeds in the control group and those treated with BR. The results showed that at all tested depths, the mesocotyl length of maize in the BR-treated group was significantly better than that in the control group (Figure 8A–C). Especially at a sowing depth of 15 cm, the emergence rate of the BR-treated group increased significantly, showing a remarkable difference compared with the control group (Figure 8A,D). These results indicate that exogenous application of BR enhances the deep-sowing tolerance of maize.

**Figure 9:**
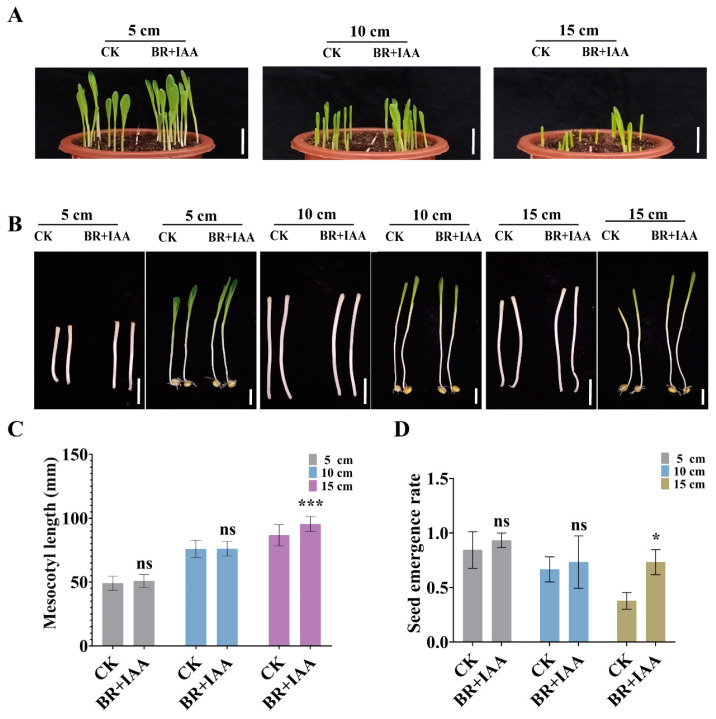
Mesocotyl length and emergence rate under the combined treatment of BR and IAA: (**A**) Ground phenotypes of Zhengdan 958 maize sown at depths of 5 cm, 10 cm, and 15 cm. Scale bar, 5 cm. CK, control group. BR + IAA, treatment with 1 μM BR and 10 μM IAA. (**B**) Mesocotyl phenotypes of Zhengdan 958 maize sown at depths of 5 cm, 10 cm, and 15 cm. Scale bar, 3 cm. CK, control group. BR, 1 μM BR treatment. (**C**) Statistics of mesocotyl length at sowing depths of 5 cm, 10 cm, and 15 cm. Data are presented as the mean ± SD (n > 14). (**D**) Statistics of emergence rate at sowing depths of 5 cm, 10 cm, and 15 cm. Statistical significance (**C**,**D**) was analyzed using Student’s *t*-test (* *p* < 0.05, *** *p* < 0.001), and ns indicates no significant difference. Data are presented as the mean ± SD (n = 3). n is the biological replicate size; a biological replicate is an independent maize mesocotyl under deep-sowing (**C**), or is a single pot (**D**).

To explore the effectiveness of exogenous co-application of BR and IAA in enhancing the deep-sowing tolerance of maize, maize seeds were sown at different depths for both the control group and the treated group. The results showed that under the sowing depth of 15 cm, the mesocotyl length of the maize seeds treated with BR and IAA was significantly longer than that of the control group (Figure 9A–C). These results indicate that the exogenous co-application of BR and IAA promoted the elongation of the maize mesocotyl and increased the emergence rate of maize at specific sowing depths (Figure 9A,D).

**Figure 10:**
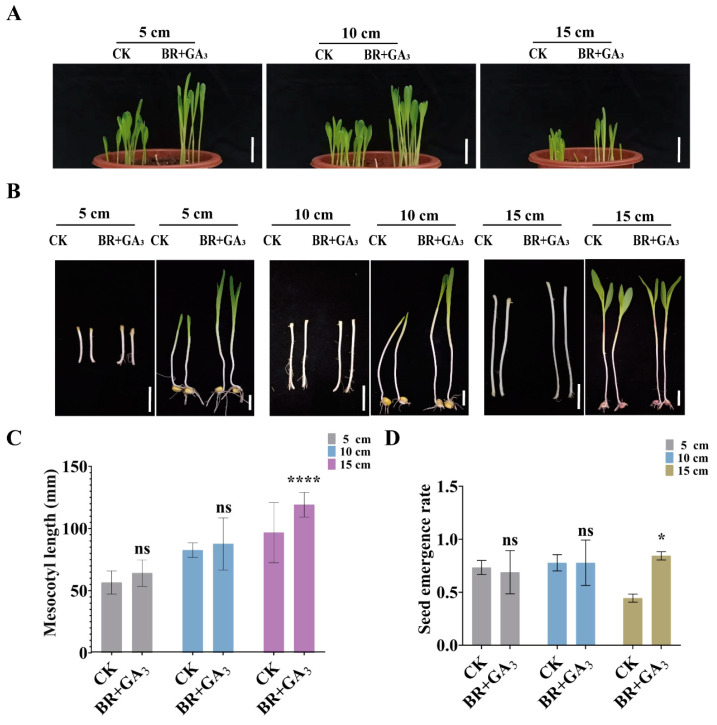
Mesocotyl length and emergence rate under the combined treatment of BR and GA_3_: (**A**) Ground phenotypes of Zhengdan 958 maize sown at depths of 5 cm, 10 cm, and 15 cm. Scale bar, 5 cm. CK, control group. BR + GA_3_, combined treatment with 1 μM BR and 1 mM GA_3_. (**B**) Mesocotyl phenotypes of Zhengdan 958 maize sown at depths of 5 cm, 10 cm, and 15 cm. Scale bar, 3 cm. CK, control group. BR, 1 μM BR treatment. (**C**) Statistics of mesocotyl length at sowing depths of 5 cm, 10 cm, and 15 cm. Data are presented as the mean ± SD (n > 14). (**D**) Statistics of emergence rate at sowing depths of 5 cm, 10 cm, and 15 cm. Statistical significance (**C**,**D**) was analyzed using Student’s *t*-test (* *p* < 0.05, **** *p* < 0.0001), with ns indicating no significant difference. n is the biological replicate size; a biological replicate is an independent maize mesocotyl under deep-sowing (**C**), or is a single pot (**D**).

To investigate the efficacy of exogenous co-application of BR and GA in enhancing maize tolerance to deep sowing, treated and untreated maize seeds were planted at varying depths. The results demonstrated that at a sowing depth of 15 cm, the mesocotyl length of BR-treated seeds was markedly longer than that of the control group (Figure 10A–C), while their emergence rate was also significantly higher (Figure 10A,D). These findings suggest that the combined application of BR and GA promotes mesocotyl elongation and improves seedling emergence in maize under specific sowing depths.

Taken together, these results consistently show that exogenous BR, either alone or in combination with IAA or GA_3_, markedly stimulates mesocotyl elongation and markedly raises the emergence rate of maize under deep-sowing conditions; the benefit is most pronounced at 15 cm sowing depth. Thus, seed treatment or soil application of BR-based hormone blends represents a simple, effective agronomic strategy for improving maize establishment in deep-sowing scenarios and can serve as a practical tool to enhance crop resilience in regions where deep planting is unavoidable.

## 4. Discussion

**Figure 11:**
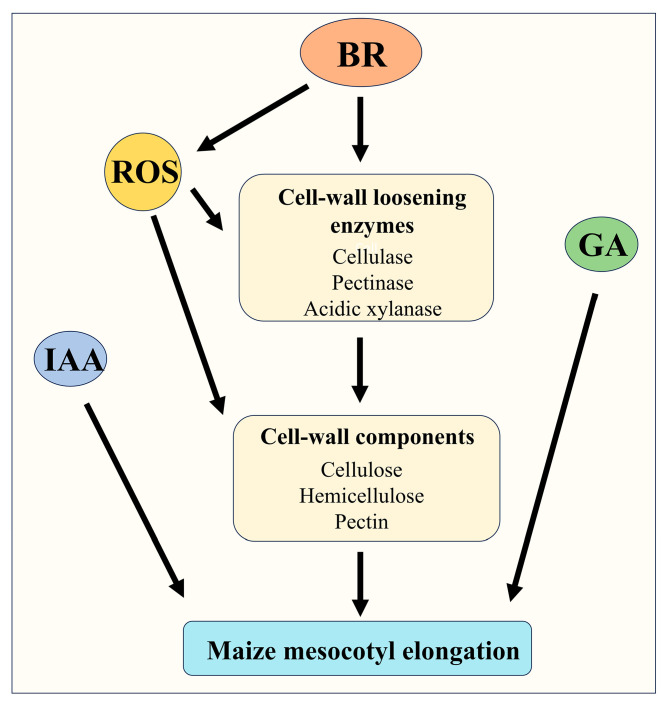
Model of BR-mediated regulation of maize mesocotyl elongation in coordination with GA and ROS.

Maize is a major food and industrial crop whose yield is strongly influenced by seedling emergence during the early growth stage. The mesocotyl elongates to push the plumule above the soil surface, enabling successful emergence [3]. Under stress conditions such as drought, sowing at greater depth can enhance seedling drought tolerance, yet it also imposes greater demands on emergence [6]. Studies have shown that mesocotyl elongation significantly promotes germination and seedling emergence [4,20,38]. BRs are plant steroid hormones that play pivotal roles in plant growth, development, and stress responses [8]. To understand how BR exerts its elongation-promoting effect on the maize mesocotyl, we dissected its influence on cell-wall architecture, the associated enzymatic, and ROS-mediated processes. Our findings indicate that BR promotes maize mesocotyl elongation through a coordinated mechanism: it downregulates major cell-wall polysaccharides (hemicellulose, cellulose, pectin), upregulates wall-modifying enzymes (cellulase, pectinase, and xylanase) activities, and shifts ROS homeostasis via SOD (Figure 11). These sequential changes reduce wall rigidity, enabling cells to expand under turgor pressure and ultimately improving seedling emergence from deep soil.

BR promotes maize mesocotyl elongation by enhancing the activities of wall-loosening enzymes (cellulase, pectinase, acidic xylanase), reducing the accumulation of cell-wall components (hemicellulose, cellulose, pectin), thereby weakening cell-wall rigidity. BR also fine-tunes ROS levels via SOD to promote cell elongation. Additionally, BR coordinated with IAA and GA to promote maize mesocotyl elongation.

ROS, once dismissed as merely toxic by-products of cellular metabolism, have now been shown to be crucial signaling molecules that play a delicate yet pivotal role in regulating plant cell elongation [27,39]. In this study, we found that H_2_O_2_ acted as a signal to regulate mesocotyl cell elongation. H_2_O_2_ directly promotes mesocotyl elongation by degrading cell-wall polysaccharides, with optimal effects at 1 mM (Figure 3B). When the concentration exceeds 1 mM, the stimulatory effect of H_2_O_2_ on mesocotyl elongation diminishes (Figure 3B). These results suggest that the effect of ROS on cell elongation is highly concentration-dependent, giving rise to an appropriate range. Within this range, ROS act as signaling molecules that induce cell-wall loosening, whereas concentrations above the optimum trigger oxidative stress and arrest growth. Further study showed that H_2_O_2_ increased the activity of cell-loosening enzymes and reduced the components of the cell wall, leading to enhanced cell elongation (Figure 5). Combined treatment with H_2_O_2_ and BR yielded greater mesocotyl elongation than either treatment alone (Figure 3); however, co-application of BR and DPI failed to augment BR’s stimulatory effect on mesocotyl elongation (Figure 4), indicating that BR’s effects are partially independent of ROS. Our findings established a concentration-tuned, dual role for H_2_O_2_ in mesocotyl growth: low-millimolar levels acted as a developmental signal that loosened the cell wall and drove elongation, whereas supra-optimal doses switched the outcome to oxidative inhibition. H_2_O_2_ and BR operated synergistically, yet BR retained the capacity to stimulate elongation through ROS-independent routes. These observations refine our understanding of how ROS and phytohormones jointly sculpt organ growth and highlight the importance of precise redox control in optimizing plant performance.

The seemingly contradictory observation that both DPI-mediated ROS suppression and exogenous H_2_O_2_ addition stimulate mesocotyl elongation actually reveals a dual, concentration-dependent role for ROS. Elongation operates within a narrow “redox window”: within this range, cell-wall loosening is maximal. When ROS fall below the lower threshold—achieved by DPI inhibition of NADPH oxidase and the consequent decline in H_2_O_2_—oxidative cross-linking in the wall is reduced, indirectly promoting cell expansion. When ROS rise above the lower threshold but remain below the upper limit, low-millimolar H_2_O_2_ acts as a signaling molecule that activates NADPH oxidase and a suite of wall-loosening enzymes, driving turgor-mediated elongation. Once ROS exceed the upper threshold, H_2_O_2_ rapidly switches from signal to inhibitor, triggering peroxidase-mediated wall stiffening and oxidative damage. BR exploits both ends of this redox rheostat: it elevates SOD activity to maintain a favorable H_2_O_2_ gradient while simultaneously engaging ROS-independent transcriptional pathways to synergistically enhance elongation (Figure 2). Thus, the “dual effect” of ROS is not paradoxical but a consequence of finely tuned, subcellular redox compartmentalization. Future work should combine tissue-specific ROS sensors with subcellular mapping to delineate how different ROS levels are spatially distributed and translated into growth responses.

Our study showed that mesocotyl elongation is tightly regulated by BR, IAA, and GA, with exogenous application of any single hormone markedly promoting mesocotyl cell elongation. Combined BR + GA treatments further enhance mesocotyl cell elongation in maize (Figure 7), suggesting overlapping regulatory pathways with GA signaling. These findings provide a novel avenue for boosting seedling emergence in arid and high-latitude maize production regions. Deep sowing is a key drought-resistance practice in maize production, and mesocotyl elongation is closely linked to successful emergence from deep soil [2]. At present, however, effective strategies for improving maize tolerance to deep sowing remain scarce. We found that BR enhances the seedling emergence ability by promoting mesocotyl elongation (Figure 8). The synergistic effect of BR with GA promoted mesocotyl elongation and increased the emergence rate at a depth of 15 cm under deep sowing conditions (Figure 10), suggesting that hormone regulation could improve the adaptability of maize to deep sowing. Our work identifies a robust, hormone-based strategy for manipulating mesocotyl growth: BR acts centrally and can be amplified by GA, resulting in a synergistic elongation response that markedly improves seedling emergence from deep sowing depths.

## 5. Conclusions

This study clarified that BR effectively promoted the longitudinal elongation of maize mesocotyl cells by weakening cell-wall rigidity, enhancing the activity of wall-loosening enzymes, and cooperatively regulating ROS homeostasis. During this process, BR markedly reduced the accumulation of hemicellulose, cellulose, and pectin while simultaneously increasing the activities of cellulase, pectinase, and acidic xylanase, rendering the cell wall more extensible. Either a moderate increase or decrease in ROS levels could act synergistically with BR to further amplify the elongation response (Figure 11). Moreover, the combined application of BR with GA showed a clear synergism on mesocotyl elongation. Deep-sowing trials demonstrated that, whether applied alone or together with IAA or GA, BR significantly lengthens the mesocotyl and elevates emergence rate, offering a simple yet highly effective technical route for safeguarding maize seedlings in arid or high-latitude regions. BR integrated hormonal and redox signals to remodel cell-wall architecture and promoted rapid mesocotyl elongation, thereby enhancing maize adaptability to deep-sowing stress and providing both theoretical insights and practical strategies for improving crop resilience under adverse conditions. These findings provide a basis for developing seed coatings or irrigation formulations that precisely modulate BR–ROS–GA crosstalk, promising robust crop establishment under deep-sowing or drought-prone field conditions.

## Data Availability

The original contributions presented in this study are included in the article/supplementary material. Further inquiries can be directed to the corresponding authors.

## Supplementary Materials

The following supporting information can be downloaded at: https://www.mdpi.com/article/10.3390/cimb47080668/s1; Figure S1: Correlation between treatment effects on mesocotyl length; Table S1: Total variance explained by principal components; Table S2: Unrotated component loading matrix.
