# Supplementary material for: Brassinosteroid Coordinates with ROS, Auxin and Gibberellin to Promote Mesocotyl Elongation and Deep-Sowing Tolerance in Maize
Source: Curr Issues Mol Biol. 2025 Aug 18;47(8):668. doi: 10.3390/cimb47080668 (PMC12384915; doi:10.3390/cimb47080668)
Supplement: Supplementary file 1 [file cimb-47-00668-s001.zip › Figure S1.pdf]

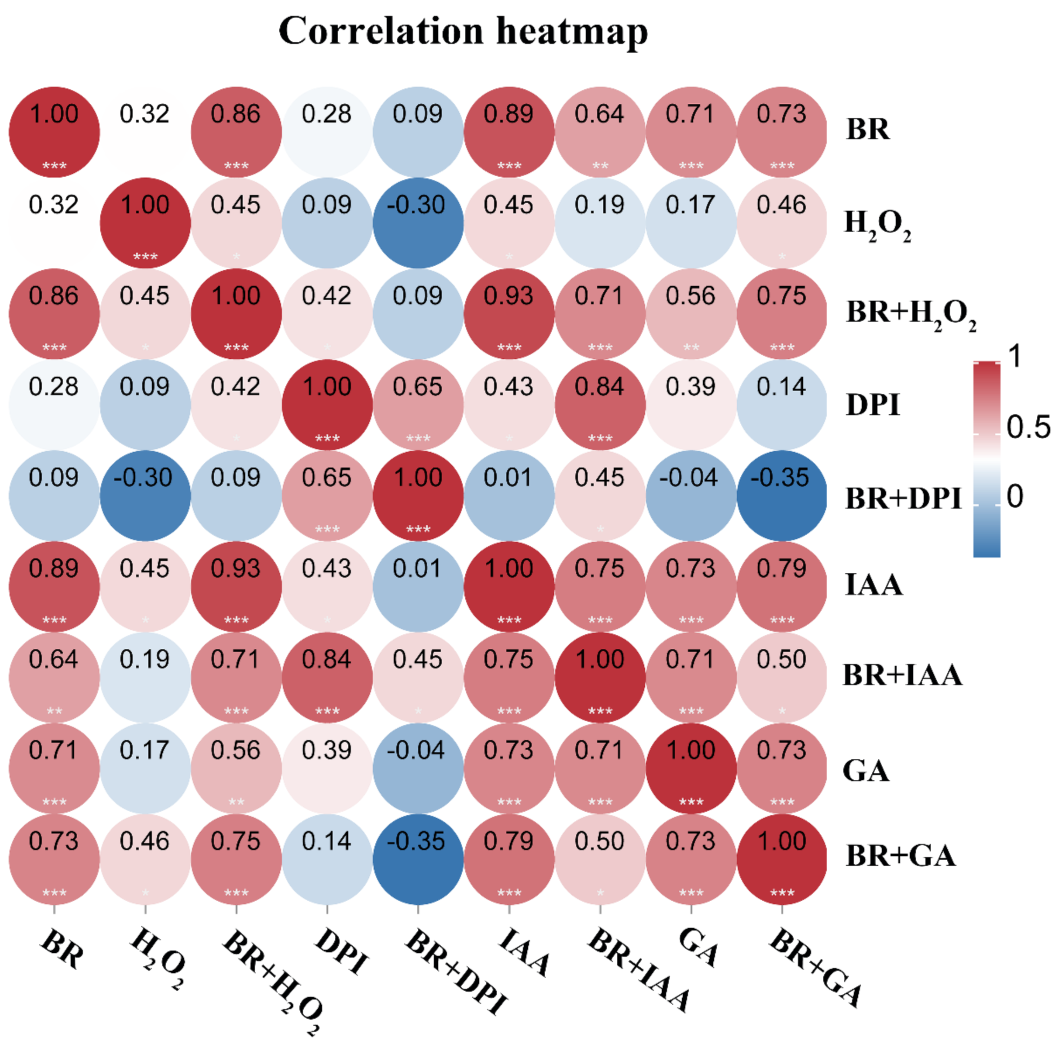

Figure S1 Correlation between treatment effects on mesocotyl length

Correlation heatmap of nine biochemical and hormonal parameters. Pearson correlation coefficients (r) are displayed in the upper triangle. Each coefficient was tested for significance with a two-tailed Student t test. Asterisks in the lower triangle indicate the adjusted significance levels: *$p < 0.05$, **$p < 0.01$, ***$p < 0.001$; non-significant coefficients are not labelled. Color intensity reflects the strength and direction of the relationship (red = positive, blue = negative).
